# Hypokalaemia-Induced Rhabdomyolysis after Treatment of Post-Kala-azar Dermal Leishmaniasis (PKDL) with High-Dose AmBisome in Bangladesh—A Case Report

**DOI:** 10.1371/journal.pntd.0002864

**Published:** 2014-06-12

**Authors:** Ulrika Marking, Margriet den Boer, Asish Kumar Das, Elshafie Mohamed Ahmed, Victoria Rollason, Be-Nazir Ahmed, Robert N. Davidson, Koert Ritmeijer

**Affiliations:** 1 Médecins Sans Frontières, Amsterdam, The Netherlands; 2 Division of Clinical Pharmacology and Toxicology, University Hospitals of Geneva, Geneva, Switzerland; 3 Communicable Disease Control, Directorate General of Health Services, Ministry of Health and Family Welfare, Dhaka, Bangladesh; 4 Department of Infection and Tropical Medicine, Northwick Park Hospital, Harrow, United Kingdom; Institut Pasteur de Tunis, Tunisia

## Introduction

Post-kala-azar dermal leishmaniasis (PKDL) is a macular, papular, and/or nodular skin rash that can appear as a sequel of visceral leishmaniasis (VL) caused by *Leishmania donovani*. In Bangladesh, it occurs in around 10% of VL patients, leading to high prevalences of 6/10,000–21/10,000 population in endemic regions [1], [2]. Over 95% of lesions are macular and cause no or little physical discomfort to patients. However, *Leishmania* parasites can been found in PKDL lesions, and there is (sparse) evidence that they are infective to sandflies [3], [4], [5]. It is generally assumed that PKDL patients form an infectious reservoir and should be treated in order to achieve disease control. There are no evidence-based treatments for PKDL. Therefore, treatment can be considered experimental, and treatment choices are “best guesses” based on good results in small clinical studies and clinical experience in the field.

Médecins Sans Frontières provides treatment for VL and PKDL in Fulbaria, a highly endemic subdistrict of Mymensingh in Bangladesh. Active case finding is undertaken, and free-of-charge short-course treatment with liposomal amphotericin B (L-AMB) (AmBisome, Gilead, United States) is provided for both VL and PKDL. L-AMB was designated by the World Health Organization (WHO) as the safest and most effective treatment for VL in the Indian subcontinent [6]. The optimal treatment for PKDL has not been established by clinical trials. Based on the safety and efficacy of L-AMB given in high total cumulative doses (50–90 mg/kg) for treatment of PKDL in small patient cohorts in East Africa [7], [8], a regimen was chosen with a total cumulative dose of 30 mg/kg, divided into six doses of 5 mg/kg L-AMB, given over a period of 3 weeks on an ambulatory basis. This dose and frequency were chosen to minimise the impact on patients' daily lives. L-AMB is known to be a safe treatment for VL in similar doses [9]. It was expected that L-AMB in this dose regimen would cause minimal adverse effects in otherwise healthy PKDL patients.

PKDL was diagnosed by clinical evaluation of lesions. More than 1,300 PKDL patients have been treated to date. Unexpectedly, we encountered hypokalaemia-induced rhabdomyolysis during or following treatment. Here, we present six confirmed cases and one presumed case of this rare adverse event that occurred in the period from October to December of 2011. After three cases of confirmed rhabdomyolysis, further enrolment of PKDL patients was stopped. Patients still under treatment were closely monitored for the occurrence of hypokalaemia.

### Presentation of Cases

#### Case 1

An 18-year-old female returned to the clinic 2 days after her fifth dose of L-AMB, unable to walk, sit up unaided, or even hold her head up due to severe muscle pain and weakness, predominantly in the proximal muscles. We were unable to perform biochemical assays. The patient improved gradually, and her symptoms resolved completely.

#### Case 2

An 11-year-old girl presented with a rapid onset of severe muscle pain and weakness in upper and lower extremities 3 days after her sixth dose of L-AMB. Serum creatinine phosphokinase (CPK) was >18,000 U/L, confirming rhabdomyolysis. Serum potassium levels were not obtained. The girl was treated with IV fluids and oral potassium supplements (potassium-rich foods), and she improved spontaneously over the next few days with a complete recovery recorded after 4 weeks.

#### Case 3

An 18-year-old male developed muscle weakness and pain a few days after receiving his sixth dose of L-AMB. Serum CPK was 11,764 U/L and serum K^+^ was 1.7 mmol/L. Urine dipstick assay was strongly positive for haemoglobin but negative for red blood cells, suggesting myoglobinuria. The patient received oral K^+^ supplementation and IV fluids and improved quickly with a complete resolution of symptoms within 1 week.

#### Case 4

Two days after her sixth dose of L-AMB, an 11-year-old girl developed severe muscle pain, weakness, arthralgia, nausea, and vomiting. Sensory examination was normal, but tendon reflexes in lower extremities were weak or absent. Investigations revealed serum CPK 12,703 U/L, K^+^ 1.7 mmol/L, and creatinine 0.5 mg/dL, suggesting rhabdomyolysis with concurrent hypokalaemia. Urine was discoloured (brown), and dipstick urine analysis was positive for haemoglobin, but urine microscopy was negative for red blood cells, suggesting myoglobinuria. The patient received IV fluids and oral K^+^ supplementation and improved with a complete resolution of symptoms within 4 weeks.

#### Case 5

A 17-year-old female developed severe muscle pain and weakness 1 day after her sixth dose of L-AMB. She was able to walk supported but could not sit up after lying down or stand up after sitting down. There was obvious muscle tenderness and a loss of tendon reflexes in upper and lower limbs. Serum CPK was 16,505 U/L, K^+^ 1.9 mmol/L and creatinine 0.6 mg/dL, suggesting rhabdomyolysis. Discoloured (brown) urine in which dipstick assay was positive for haemoglobin but urine microscopy negative for red blood cells suggested myoglobinuria. The patient received oral K^+^ supplementation and IV fluids and recovered fully within 2 weeks.

#### Case 6

A 13-year-old girl developed hypokalaemia (serum K^+^ 2.6 mmol/l) at her sixth dose of L-AMB. Urine dipstick was positive for haemoglobin but urine microscopy negative for red cells, suggesting myoglobinuria. Upon questioning, she admitted experiencing muscle pain, nausea, and vomiting. On examination, there was obvious muscle tenderness. She received oral K^+^ supplementation and IV fluids. Her serum CPK obtained 3 days after start of K^+^ supplementation was 2,233 U/l and creatinine 0.5 mg/dl. Her symptoms resolved completely within 2 weeks.

#### Case 7

A 13-year-old female gradually developed moderate hypokalaemia, with a nadir serum K^+^ of 2.8 mmol/L, 1 week after receiving her sixth dose of L-AMB. On examination, there was muscle tenderness in thighs and muscle pain on exertion. Serum CPK rose from 140 U/L at this point to 2,548 U/L 1 week later. Serum creatinine remained 0.5–0.6 mg/dL. She received K^+^ supplementation from her fourth dose of L-AMB on, and her symptoms resolved completely one week after onset.

All seven patients were followed up with history, clinical examination, and testing of serum CPK, K^+^, Na+, creatinine, urea, liver enzymes, and urine dipstick 2–4 months later. These revealed no relevant pathology, and in all cases, CPK returned to normal levels, and symptoms completely resolved.

## Case Discussions

Six cases of confirmed and one presumed case of clinical rhabdomyolysis post-PKDL treatment with L-AMB 30 mg/kg were diagnosed over a period of 3 months. Three patients had rhabdomyolysis accompanying severe hypokalaemia (serum K+ <2.5 mmol/L), and in two further cases, hypokalaemia was moderate (2.5–3.0 mmol/L), although it is possible that the lowest K+ levels were not recorded. Notably, in both these cases, hypokalaemia occurred a few days before serum CPK was significantly elevated. Five of six were females aged 11–17 years. Apart from PKDL, none had any significant comorbidity, concomitant use of drugs, or other cofactors. Positivity of urine haemoglobin assay was considered to be due to myoglobinuria, supported by the concomitant elevation of CPK and absence of red blood cells in urine. Haemolysis was not suspected, and there were no clinical signs of anaemia. Haemoglobin levels in blood were not obtained.

A retrospective search was done for symptoms consistent with rhabdomyolysis in the records of all PKDL patients (n = 1,292) who had received L-AMB, and it found a further 19 patients with symptoms consistent with rhabdomyolysis. Among these 19 patients diagnosed in retrospect, 18 were female and 15 aged 11–26 years. No patients <11 years old developed rhabdomyolysis. In all cases, there was a clear temporal relationship between onset of the myalgia or muscle weakness and treatment with L-AMB and between the resolution of symptoms and ending L-AMB. No indication was found that another diagnosis or another drug was implicated in either the seven directly observed cases above or among the 19 possible cases of rhabdomyolysis that were diagnosed retrospectively. Pharmacovigilance analysis of the patient files by the Geneva University Hospital considered a causal link to the L-AMB treatment probable in 23 and possible in three of the 26 of the PKDL patients. PKDL patients are not thought to have disease of the muscles or of any organ system other than the skin. L-AMB was developed primarily to reduce nephrotoxicity commonly seen with conventional amphotericin B deoxycholate (c-AMB). Compared to c-AMB, L-AMB has a greatly improved safety profile [10]; the encapsulation of amphotericin B in liposomes leads to decreased glomerular filtration and an altered distribution with low plasma levels of free amphotericin B, increasing its efficacy and significantly reducing nephrotoxicity [11], [12].

Both nephrotoxicity and hypokalaemia are dose-dependent adverse reactions to L-AMB treatment [13], [14]. Hypokalaemia is commonly seen after both c-AMB and L-AMB administration; however, it is less frequent with L-AMB [10], [15]. In three large trials establishing safety and tolerance of L-AMB, hypokalaemia was seen in 22–43% of patients treated with 3–15 mg/kg/day of L-AMB for up to 83 days (maximum cumulative dose 972 mg/kg) [10], [15]–[17]. Severe hypokalaemia <2.5 mmol/L was reported in one study, affecting 7% of patients treated with 3 mg/kg/day for a mean of 11±9 days. Overall, severe hypokalaemia is rare, but it is reasonable to assume that study participants received potassium supplementation when necessary.

Hypokalaemia-induced rhabdomyolysis caused by c-AMB has been seldom reported and only in paediatric patients [18]. The true incidence of post-L-AMB rhabdomyolysis is unknown. Rhabdomyolysis was not a known side effect of L-AMB until it was occasionally reported in post–marketing surveillance and remained very rare: in 21 years of experience, only 8–9 cases have been reported from Japan (incidence 1∶100,000) (verbal communication, Gilead). In 2012, rhabdomyolysis was approved by the US Food and Drug Administration (FDA) for inclusion as an infrequent adverse effect in the AmBisome package insert. The FDA reports accessed through FDA Freedom of Information Department reveal 27 unique cases of rhabdomyolysis after AmBisome, including 14 of the cases reported in this paper above. However, no details were shared, and diagnoses may be uncertain. In principle, medical professionals are required to report all adverse events related to a licensed drug. In practice, this is seldom done; moreover, it is at the discretion of the professional to decide whether an adverse event is related to the drug. Hypokalaemia-induced rhabdomyolysis can prove challenging to diagnose, as muscle lysis will lead to an immediate and often significant increase in serum potassium levels, masking the aetiology. Moreover, one can argue mild cases of rhabdomyolysis may go unnoticed in the severely ill, bed-bound patients with fungal infections who are the typical recipients of L-AMB (as opposed to our otherwise healthy patient group).

A possible explanation for unexpected adverse events with a well-known drug is a drug-quality problem; this was excluded by reanalysis of a sample of L-AMB by Gilead. One vial of unreconstituted AmBisome from the batch used in the clinic at the time when the above cases were treated was randomly selected and transported, in temperatures below 25°C, to Gilead's laboratory in the US. The sterile water and 5% dextrose solution used for reconstitution and infusion of AmBisome had both undergone thorough testing before the start of the program, and the same manufacturer was employed throughout the program. Exposure of L-AMB to temperatures over 30°C will increase its toxicity because the liposomes will start to deteriorate and release free amphotericin B. The Médecins Sans Frontières (MSF) team in Dhaka has confirmed that storage and transport were carried out in temperatures below 25°C. Temperatures are recorded at all times, and recording devices' logs were double-checked.

Hypokalaemia is a well-established cause of muscle-cell necrosis and rhabdomyolysis. However, it is not clear why our patients reacted with such severe hypokalaemia to L-AMB treatment. It is possible that the events are related to the use of L-AMB in a chronically malnourished population. However, apart from clinical studies, safety data from routine use of L-AMB in resource-limited settings is very limited, as no systematic post-marketing pharmacovigilance surveillance has been done in Asia and Africa, except in India, where MSF implemented L-AMB for VL in a total dose of 20 mg/kg, administered as 5 mg/kg doses on days 0, 1, 4, and 9. No serious side effects were observed in a cohort of 2,510 patients [19], apart from a non-life-threatening swelling of the lips. However, electrolytes and creatinine were not routinely measured. In our patients, toxicity generally occurred at the fifth or sixth dose of L-AMB (25 mg/kg total dose and 30 mg/kg total dose, respectively), suggesting a cumulative effect. It should be noted that in 20 clinical trials of L-AMB in VL a total of 2,293 individuals have received total doses of 3.75–76 mg per kg and daily doses of 1–15 mg per kg; no cases of rhabdomyolysis and no cases of serum K+ <2 mmol/L were reported [19]–[38]. Mild hypokalaemia was rarely reported but not actively monitored in all trials.

Among our patients with rhabdomyolysis, six of seven were adolescent girls, a pattern that is also seen among the possible cases diagnosed in retrospect. Why this group is more susceptible than others is unclear. Micronutrient deficiency might provide an explanation and has been found among adolescent girls in Bangladesh in several studies [39]–[42]. Magnesium deficiency aggravates hypokalaemia, which often becomes refractory to treatment. Amphotericin B, also in liposomal form, induces magnesium wasting [17], [43], further worsening a possible deficiency. It has been suggested that the high prevalence of eclampsia among young Bangladeshi women is explained by high rates of magnesium deficiency [44].

Because PKDL in Bangladesh is a non-life-threatening condition that generally does not cause physical impairment and because treatment is mainly provided for public health reasons, the risk/benefit ratio of treatment for individual PKDL patients needs to be extremely low, using a very safe treatment. Because of the observed occurrence of life-threatening side effects, a 30 mg/kg total dose L-AMB regimen is not recommended as routine treatment for PKDL in Bangladesh. Further research on the effectiveness and safety of a lower dose L-AMB regimen is currently planned in order to establish an effective, safe, and acceptable treatment for PKDL that does not require strict clinical and laboratory monitoring and that can be adopted in the National Kala Azar Elimination Programme in Bangladesh. Until more is known about the safety of L-AMB in this particular setting, it may be advisable to monitor serum potassium levels in patients receiving L-AMB in cumulative doses of 20 mg/kg or more.

### Consent for Publication

All patients or their caretakers have given written informed consent for publication.

**Learning Points**

- Life-threatening hypokalaemia-induced rhabdomyolysis may occur in PKDL patients who otherwise seem healthy during treatment with high-dose liposomal amphotericin B.
- The safety profile of liposomal amphotericin B can vary between populations, possibly depending on factors like locally prevailing nutritional and micronutrient status, background concomitant drug use, or other host-related predisposing factors.
- Introduction of a safe and well-known drug in a new context requires strict safety monitoring to detect unexpected serious side effects.
